# Characteristics of Livestock Husbandry and Management Practice in the Central Dry Zone of Myanmar

**DOI:** 10.1007/s11250-018-1738-9

**Published:** 2018-10-30

**Authors:** Tu Tu Zaw Win, Angus Campbell, Ricardo J. Soares Magalhães, Kyaw Naing Oo, Joerg Henning

**Affiliations:** 1 The School of Veterinary Science, The University of Queensland, Gatton, Australia; 2 Faculty of Veterinary & Agricultural Sciences, The University of Melbourne, Melbourne, Australia; 3 Children’s Health and Environment Program, Child Health Research Centre, The University of Queensland, South Brisbane, QLD 4101 Australia; 4 Livestock Breeding and Veterinary Department, The Ministry of Agriculture, Livestock and Irrigation, Nay Pyi Taw, Myanmar; 5 The School of Veterinary and Life Sciences, College of Veterinary Medicine, Murdoch University, Perth, Western Australia 6150 Australia

**Keywords:** Livestock, Husbandry practice, Multispecies, Herd size, Purpose

## Abstract

The central dry zone of Myanmar is the area with the highest density of small-scale livestock farmers under harsh environmental condition. In this study, we describe and quantify ownership patterns for various livestock species and characterised management and husbandry practices of small-scale farmers. In addition, we identify the husbandry factors associated with selected outcome indicators, ‘herd or flock size’ and ‘purpose of rearing’. A total of 613 livestock farmers in 40 villages were interviewed. Multispecies rearing was common with 51.7% of farmers rearing more than one livestock species. Rearing animals to be sold as adults for slaughter (meat production) was more common for small ruminants (98.1%) and chickens (99.8%) compared to cattle (69.8%). Larger cattle herds were more likely to practice grazing (*p* < 0.001) and to employ labour from outside the household to manage cattle than medium or small herds (*p* = 0.03). Patterns of grazing differed significantly between seasons (*p* < 0.01) for cattle, but not for small ruminants and village chicken. Overall, multispecies rearing and species-specific husbandry practices are used to raise livestock under harsh environmental conditions. Our results reveal that herd/flock size and purpose of rearing across different livestock species were significantly associated with feeding and housing practices and experience of farmers.

**Electronic supplementary material:**

The online version of this article (10.1007/s11250-018-1738-9) contains supplementary material, which is available to authorized users.

## Introduction

Typically, descriptions of livestock production systems concentrate on one species of animal, although households in developing countries might keep multiple species and interrelationships in the management are likely to exist. In addition, livestock production in developing countries is often constrained by poor husbandry, inadequate housing and poor breeding, health and biosecurity practices (Conan et al. 2013; Gillette 2013; Homann et al. 2007; Nampanya et al. 2012). Thus, in resource poor households that keep multiple livestock species, investments into feeding and housing need to be spread across various livestock species. It has been shown that farmers’ income is largely influenced by herd size (Bailey et al. 1997; Maltsoglou and Rapsomanikis 2005; McPeak 2004; Oleggini et al. 2001), and understanding factors that impact on herd size, in particular in multispecies households, is critical for rural livestock development (Kaimba et al. 2011; Loibooki et al. 2002). In addition, some livestock species are raised predominantly for sale, while others are more important for home consumption or to support other agriculture activities such as the use of cattle for draught power (Alam 1997; Kristjanson et al. 2004a; Moll 2005; Yamamoto 2004). Thus, understanding husbandry factors that influence the multiple purposes of livestock rearing is essential in order to work with livestock farmers on improvement of livestock production.

Unfortunately, little is known about livestock production in Myanmar, despite its great importance in Southeast Asia: approximately 13 million cattle, 3 million sheep and goats and 135 million poultry were kept in Myanmar in 2009 (OIE 2009). Livestock in Myanmar is mainly reared on ‘backyard farms’, with feeding provided in traditional ways such as grazing common in fallow areas within and around villages or scavenging in the village environment and utilising standing crop residues and by-products (Devendra and Thomas 2002a, b; Devendra et al. 1997; Henning et al. 2007; Oo 2010). The central dry zone (CDZ) is a major hub for crop and livestock production with almost 50% of Myanmar’s livestock population being reared in this area. Even though livestock production is considered to be a major income source for farmers in the CDZ, there is an eminent lack of information on livestock husbandry practices, nutrition, animal health problems, the socio-economic impact of livestock production and the current trading system.

In this study, we describe ownership patterns for various livestock species and characterise management and husbandry practices of small-scale farmers. We then select ‘herd or flock size’ as a measure not only describing the ‘wealth’ of farmers but also reflecting the success of livestock production and identifying factors of management and husbandry practices impacting on establishing herd or flock sizes. We also explore factors that impact on ‘purposes of livestock rearing’ because it describes the diversity of benefits that can be derived from livestock rearing.

## Methods

### Study design

**Fig. 1:**
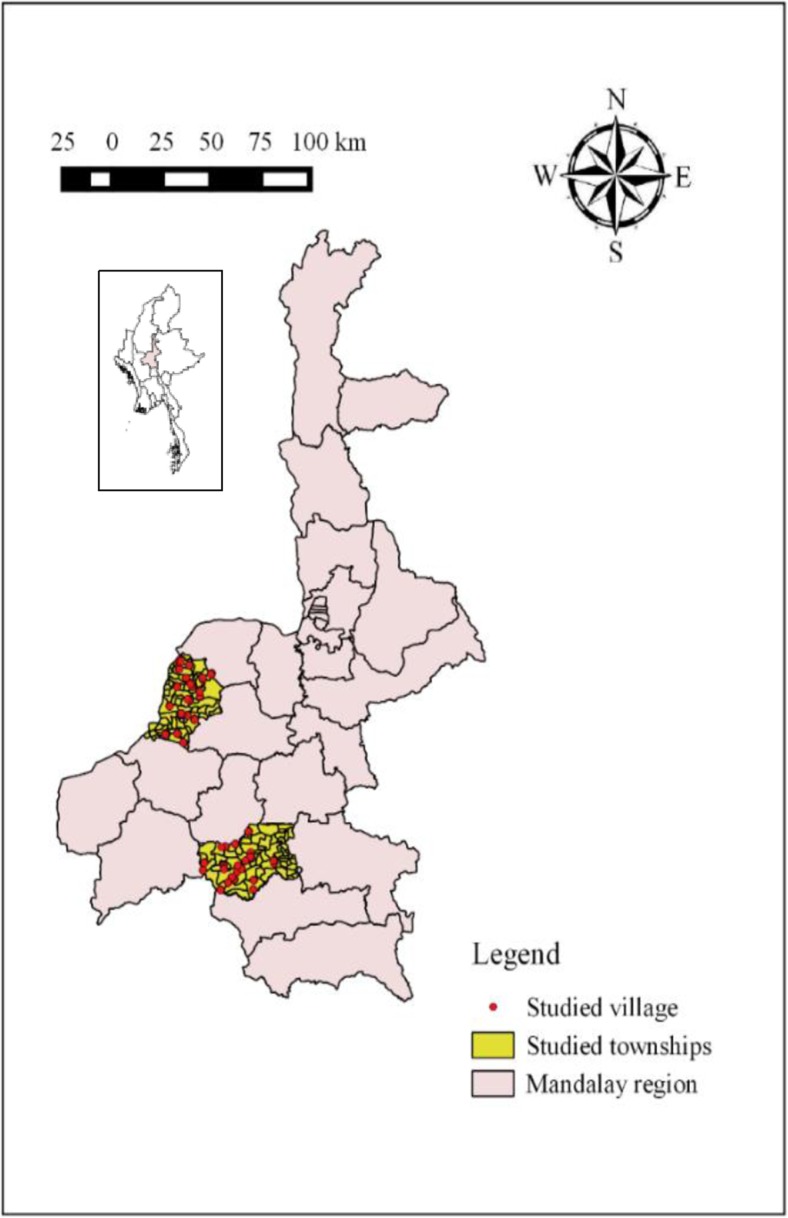
Map of Myanmar highlighting the Mandalay region where research on multispecies livestock rearing was conducted between November and December 2014. The two study townships (Meikhtila and Myingyan) are shown in yellow, and the study villages are shown in red

The cross-sectional study involving small-scale farming households owning different livestock species was conducted in the CDZ of Myanmar. The data collection was conducted between November and December 2014 in two administrative areas (‘townships’), Myingyan and Meikhtila, of the CDZ (Fig. 1). These two townships were identified as being representative for CDZ livestock holdings, production systems and the environment by a livestock research project (AH/2011/054) funded by Australian Centre for International Agriculture Research (ACIAR) (ACIAR 2014).

### Sample size calculation and selection of sampling units

A two-stage sampling approach was used, with villages (‘clusters’) and households comprising the two sampling stages. The proportion of farm income generated from livestock production was used as the outcome of interest for the sample size calculations, conservatively assumed to be 50%, with within- and between-cluster variances of ± 10% and ± 2.5%, respectively. he low between-cluster variance reflected the very similar ecological conditions resulting in similar income generation from livestock production across villages in the CDZ. The estimated sample size was 20 households per village and 38 villages across the two townships, assuming that the proportion of farmers in a village deriving at least half of their income from livestock production was 0.7, a population of 400 villages per township and approximately 200 households per village based on livestock statistics data compiled by the Myanmar Livestock, Breeding and Veterinary Department (LBVD) (LBVD 2014). The precision of the estimate was set to ± 5% with a 95% confidence interval. Lists of villages were provided by LBVD. In order to select villages, a probability proportional to size sampling strategy was used (http://epitools.ausvet.com.au/content.php?page=2StagePrevalence1), giving larger villages a greater probability of being selected; a total of 40 villages were selected in each township (20 villages to be selected and 20 potential replacement villages). Within selected villages, lists of households for each of the three major livestock species (cattle, small ruminates and village chickens) were provided by village headmen. Selected villages were replaced if they had insufficient households with the three livestock species of interest or if farmers were not willing to participate in the study. Seven households from each livestock ownership list were randomly selected, providing a total of 21 households per village. If farmers in selected households refused to participate in the study, replacement households were randomly selected. Sample size calculations and random sampling were performed using the Survey Toolbox modules: sample size for two-stage prevalence survey, random sampling from a sampling frame (http://epitools.ausvet.com.au/content.php? page=Random Sampling1) and random sampling of animals, respectively (http://epitools.ausvet.com.au/content.php?page=RandomSampling2) (Sergeant 2014).

### Livestock husbandry questionnaire and data collection

The ethical approval for conducting the interviews with farmers was provided by the University of Queensland Human Research Ethics Committee (approval number #2014001425). A questionnaire was used to collect demographic details of farmers, information on herd structure, husbandry practices and purpose of rearing. The questionnaire was developed in English and translated into the local language (Myanmar/Burmese). The questionnaire was piloted in six households owning multiple livestock species (cattle, goats and chickens) across two villages—one relatively poorer and one more affluent—in Meikhtila Township. After the pilot testing, a total number of 32 questions were modified. Questions on home asset scores and feeding and housing were adjusted to be more relevant to the local conditions and to improve farmers’ understanding of the questions. The final questionnaire had 34 questions for each livestock ownership groups, and the average duration of an interview was approximately 1 hour. The survey was conducted from November 2014 to January 2015. The interviews were conducted by seven enumerators, comprising of Myanmar University of Veterinary Science postgraduate students and LBVD staff. All enumerators were trained in the survey and interviewing techniques before the survey commenced.

### Categorisation of variables

The number of animals kept per herd or flock was examined by tercile analysis, and the 33rd, 66th and 100th percentiles were used to describe herd/flock sizes. Herds/flocks were classified into three sizes (small, medium, large), corresponding to these terciles for each livestock species: cattle herds—small (1–3 head), medium (4–6) and large (> 6); small ruminants flocks—small (1–20), medium (21–40) and large (> 40); and village chicken flocks—small (1–7), medium (8–14) and large (> 14).

Purposes of cattle rearing were specified by farmers as ‘meat production (i.e. sale of adult animals for slaughter)’, ‘milk production’, ‘draught power’, ‘breeding and sale of offspring’ and ‘manure used for fertilizer’. Cattle rearing for meat production, ‘breeding’ and/or milk production was combined into the category of ‘cash commodity’ purpose; cattle rearing for draught power and ‘manure for fertilizer’ was into the category of ‘agriculture focus’ purpose; and the combination of any these two categories was regarded as ‘multipurpose’ cattle rearing. As chickens and chicken products (eggs) and small ruminants and theirs products (milk) were only used by farmers for sale and home consumption, we were not able to categorise purposes of livestock production for these two livestock species a into separate categories.

### Statistical analysis

We considered seven different types of livestock ownership: rearing either cattle, small ruminants or village chickens alone, rearing combinations of two livestock species or rearing all three livestock species together.

Data checking and validation was conducted by using NVivo Pro 11. Data were analysed using survey design commands in Stata 14.0 (Stata Statistical Software, College Station, Stata Corporation 2015) to account for the two-stage study design, with sampling weights, sampling strata (townships) and clustering effects (villages) specified beforehand (Deaton 1997; Nathan and Holt 1980). The primary sampling units (PSUs) were villages within the townships, and the secondary sampling units (SSUs) were households within these villages. Sampling weights for the household and village level represented the inverse of the probability of being sampled (StataCorp LP 2014). Taylor linearization was used for variance estimation (VCE) (Cochran 1977; Wolter 2007), with a finite population correction (FPC) used for each sampling level by specifying the total number of villages and the total number of households. Two different sampling weights were used for the household and village level, representing the reverse of the probability of being sampled. The PSUs (villages) were also stratified into two strata (townships), addressing decreasing variability as sampled villages are more homogenous within the strata than between the strata (Heeringa et al. 2010; Levy and Lemeshow 2013; Skinner et al. 1989). Finite population corrections (FPC) were applied for each level, representing the number of total villages and households in the studied areas. This allowed accounting for the reduction in variance by comparing sampling without population replacement from a finite population with sampling with replacement from the same population (Cochran 1977).

The proportion of farmers having different herd/flock sizes categories (small, medium, large) and the proportion of framers conducting different management practices (e.g. housing, feeding and breeding practices) were compared between livestock ownership groups using the Pearson *χ*^2^ statistics, which was converted into *F*-statistics accounting for the survey design (Koch et al. 1975; Rao and Scott 1984). In addition, the proportion of farmers conducting seasonal feeding for each livestock species was also compared using the survey design-converted *F*-statistic.

To identify factors that influence herd/flock size (low-medium-high) and the purpose of livestock rearing ordinal logistic regression and multinomial logistic regession models were built for each livestock enterprise (cattle, small ruminants and chickens). The proportional odds ratio assumption for the use of ordinal regression was assessed using the likelihood ratio test (-omodel- command in STATA) and the Brant test (-brant- command in STATA) (Agresti and Kateri 2011; Long and Freese 2006; Paxton 1999; Sloane and Morgan 1996). A non-significant result would indicate thet parallel regression or proportional odds assumption is not violated (IRDE 2016). Similarly, nominal regression was used to identify livestock management practices that were associated with purpose of cattle rearing.

Management factors significant at *p* < 0.05 in the univariable analyses were included in the multivariable analyses in an initial forward selection and then backward elimination building procedure until all variables were signficant at *p* < 0.05. The Wald test was used to assess the joint signficance of varibales with more than two levels. The final, best-fitting model was selected as the one with the lowest Akaike Information Criterion (AIC).

## Results

### Ownership patterns

It was aimed to collect data from seven households owning each of the three livestock species in each of the 40 villages, representing 280 households for each species and 840 households altogether. However, many of the households, selected from the sampling frame of cattle, small ruminant or village chicken owners, also kept other livestock species, and we also collected data for these additional species in the same household. As a result, fewer individual households were surveyed, with a total 613 household owners were interviewed, with cattle being raised in 382, small ruminants in 303 and village chicken in 327 households.

Men comprised 49.8% (95% CI 44.2–55.4) of the interviewees, and 50.2% (95% CI 44.6–55.9) were women. The mean age of the respondents was 47 (range 12–84) years.

**Fig. 2:**
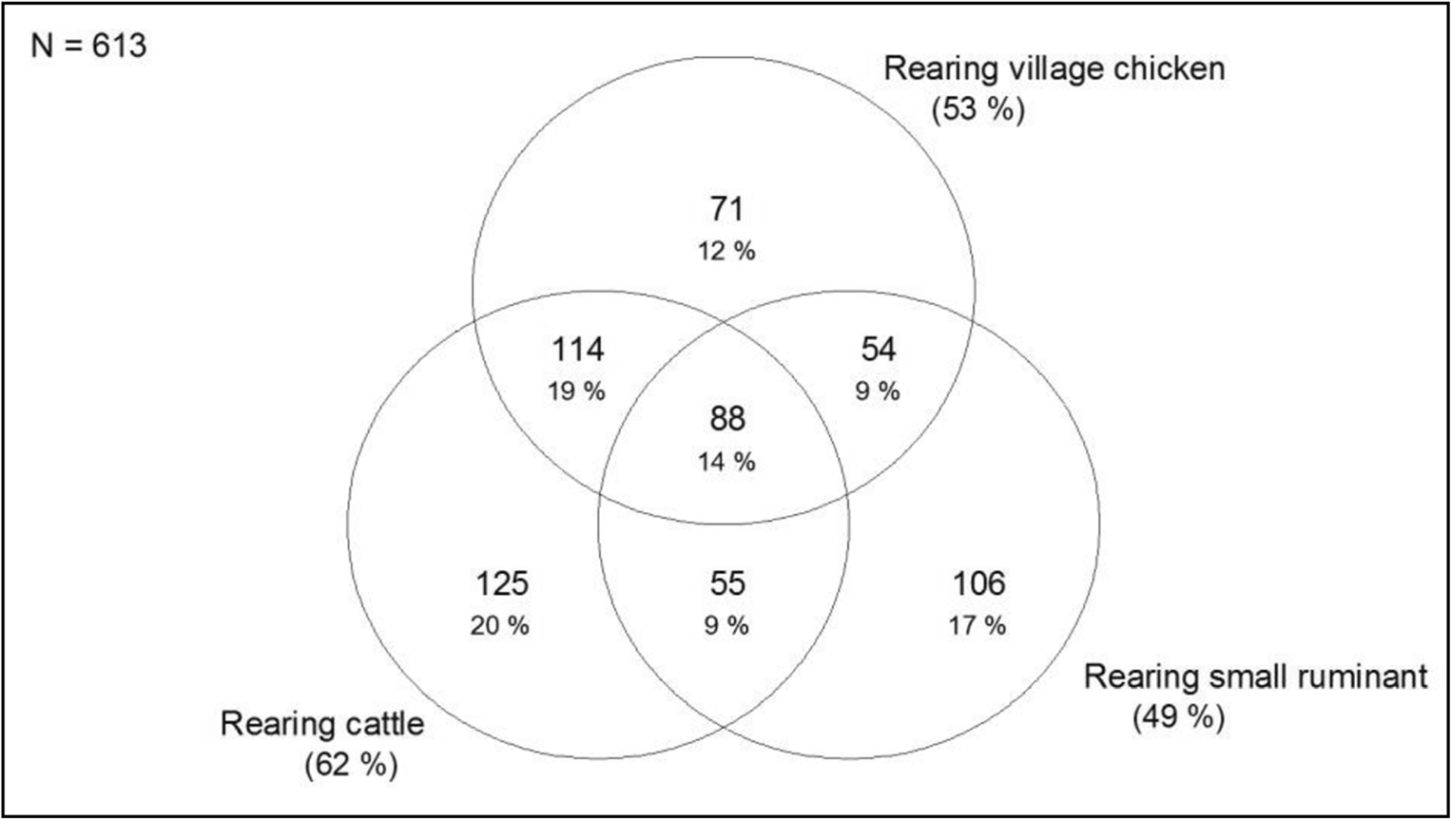
Proportion of farmers raising single species or combinations of livestock species in the CDZ of Myanmar (N cattle farmers 382; N small ruminant farmers 303; N village chicken farmers 327)

Survey households (62.3%) owned cattle, followed by village chicken (53.3% of 613 households) and small ruminants(49.4% of 613 households). Mixed livestock rearing was common, with 311 (51.7% of 613 households) households rearing more than one livestock species (Fig. 2). Of the 613 households, 19.6% of households had cattle only; 18.9% of households kept cattle and village chicken; 16.8% of households raised small ruminant only; 15.5% of households raised cattle, small ruminant and village chicken together; 12.2% of households had village chicken only; 9.2% of households had cattle and small ruminants; and 7.8% of households raised small ruminant and village chicken.

Approximately three quarters of the cattle and two thirds of village chicken owners raised these species for more than 10 years, while the majority of small ruminant farmers (in particular sheep farmers) had less than 5 years’ experience (Supplementary Table 1).

### Herd or flock size

**Fig. 3:**
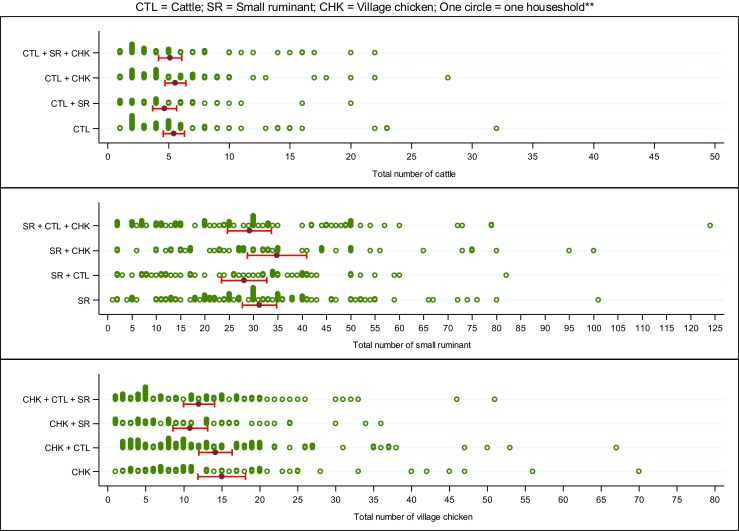
Distribution of cattle (CTL), small ruminates (SR) and village chicken (CHK) herd or flock sizes by livestock-ownership groups in the CDZ of Myanmar. Red horizontal bar indicate the mean herd/flock size with its 95% confident interval

Herd/flock sizes varied across different livestock ownership categories as shown in Fig. 3. The median herd size for cattle was 4 animals (IQR 2–7), comprising of one male calf (range 1–5), one female calf (range 1–10), one cow (range 1–30) and one bull (range 1–23). For small ruminants, the median size was 30 (IQR 15–41), comprising of three (range 1–30) male offspring, four (range 1–30) female offspring, 17 (range 1–65) adult females and two (range 1–50) adult males. The median village chicken flock size was 10 (IQR 5–18), comprising seven (range 1–400) chicks, two (range 1–30) hens and one (range 1–17) rooster. There was no significant difference in the proportion of households with ‘small, ‘medium’ or ‘large’ herds/flocks of cattle, small ruminants or village chickens across the different livestock ownership groups (*p* = 0.34, 0.51 and 0.79 for cattle, small ruminant and village chicken ownership groups, respectively; Table 1).

**Table 1:** Husbandry practices conducted by farmers owning cattle, small ruminant or village chicken singly or in combination with other species in the CDZ of Myanmar

Type of livestock enterprise		Provision of shelter (%)	Practice grazing (%)	Provision of any supplementary feed at home (%)	Herd/flock size			
					Small (%)	Medium (%)	Large (%)	Median
Husbandry practice of cattle in households owning cattle singly or with other livestock species								
CTL	Only (*N* = 125)	91.4	71.6	90.8	38.3	39.4	22.3	4
	+ SR (*N* = 55)	74.2	81.3	71.4	38.5	38.7	22.9	4
	+ CHK (*N* = 114)	77.6	78.5	84.6	37.3	29.8	32.9	4
	+ SR + CHK (*N* = 88)	79.6	77.0	83.0	53.5	23.1	23.3	3.5
Husbandry practice of small ruminant in households owning small ruminant singly or with other livestock species								
SR	Only (*N* = 106)	96.1*	98.8	14.5	24.0	50.2	25.8	30
	+ CTL (*N* = 55)	87.35*	97.1	10.2	35.4	42.0	22.6	29
	+ CHK (*N* = 54)	97.6*	0.0	10.4	20.3	45.7	34.0	30
	+ CTL + CHK (*N* = 88)	89.8*	97.7	14.5	33.9	37.7	28.4	26
Husbandry practice of village chicken in households owning village chicken singly or with other livestock species								
CHK	Only (*N* = 71)	10.0	94.1	98.1	32.6	31.2	36.2	11
	+ CTL (*N* = 114)	10.6	88.7	92.7	32.3	36.7	31.0	10
	+ SR (*N* = 54)	19.3	90.7	98.4	32.2	34.1	33.7	9
	+ CTL + SR (*N* = 88)	12.8	82.7	98.5	44.0	24.5	31.5	11

*CTL* cattle, *SR* small ruminant, *CHK* village chicken

*Significant at *p* < 0.05 for comparison between livestock ownership groups

### Purpose of raising livestock

Livestock species were reared for different purposes. The majority of cattle farmers conducted cattle raising for multiple purposes (50.8%), followed by raising them for draught power for crop production (33.5%), while rearing cattle for sale was less common (15.7%). Manure from cattle was used by 56.7% of cattle-rearing households as fertiliser. Breeding small ruminants for the sale of offspring (88.1% of 303 small ruminant farmers) was more common than for cattle (74.2% of 382 cattle farmers). About one third of households kept cattle (31.6%) or small ruminants (28.6%) for milk production. Cattle and small ruminats were not raised for home consumption. Rearing animals to be sold as adults for slaugter (meat production) was more common for small ruminants (98.1%) and chickens (99.8%) compared to cattle (69.8%).Village chickens were predominately raised for the cash sale of live birds (77.2% of 327 households), followed by home consumption (22.6%) and cockfighting (0.2%).

### Livestock husbandry characteristics

Raising cattle, small ruminants or village chickens alone, with one other livestock species or all three livestock species together, did not influence the nutritional management (i.e. grazing practices, provision of supplementray feed and water). Similarity, grazing was common for both cattle (~ 70% of 382) and small ruminants (~ 90% of 303), whereas provision of cut and carry grass was more frequently conducted for cattle (~ 50%) compared to small ruminants (~ 2%). Patterns of cattle grazing differed significantly between seasons (*p* < 0.01). Seventy-four percent of cattle herds were taken out for grazing in the rainy season (June–October) and winter (November–February), whereas only 62.0% of herds grazed in the summer months (March–May; Table 2). Providing supplementary feed to cattle was more common (> 50% of HH) during summer and then decreased (< 50%) in the winter and rainy seasons. In contrast, no seasonal differences were observed for small ruminant grazing, with approximately 98.0% of small ruminant flocks grazing in summer, the rainy season and winter alike. Similarly, there were no seasonal differences in nutritional management of village chickens, with 90.0% of village chicken flocks scavenging in all three seasons of the year. Additional feeds such as rice (90.0%), food scraps (48.0%), maize/sorghum (25.0%) and broken rice (10.0%) were provided. Wells were the most common source of drinking water for all species (70.0–80.0%). No water was provided at home to approximately 5% of ruminant herds and 13% village chicken flocks (Table 2).

**Table 2:** Seasonal variation of feeding and watering practices conducted by cattle, small ruminant and village chicken farmers in the CDZ of Myanmar

No.	Feeding practice	Categories	Cattle		Small ruminants		Village chickens	
			N	Proportion with 95% CI	N	Proportion with 95% CI	N	Proportion with 95% CI
1.	Use of grazing areas	Summer	382	62.1* (54.2-69.4)	303	98.4 (95.2-99.5)	N/A	
		Rainy season	382	74.4 (66.8-80.8)	303	98.4 (95.2-99.5)		
		Winter	382	73.2 (66.0-79.3)	303	98.4 (95.2-99.5)		
2.	Provision of cut and carry local fodder grass	Summer	382	29.1* (22.9-36.1)	303	1.6 (0.5-5.4) to	N/A	
		Rainy season	382	78.1 (71.8-83.3)	303	1.6 (0.5-5.4)		
		Winter	382	74.2 (67.8-79.7)	303	1.4 (0.3-5.4)		
3.	Provision of rice straw	Summer	382	47.2* (38.2-56.4)	303	1.9 (0.6-5.8)	N/A	
		Rainy season	382	13.9 (9.9-19.3)	303	1.9 (0.6-5.8)		
		Winter	382	12.4 (8.7-17.4)	303	1.9 (0.6-5.8)		
4.	Provision of crop residue**	Summer	382	71.3* (66.3-75.9)	303	11.7 (6.6-20.0)	N/A	
		Rainy season	382	41.6 (35.0-48.4)	303	10.8 (6.2-18.4)		
		Winter	382	43.5 (37.6-49.7)	303	10.5 (6.0-17.8)		
5.	Provision of groundnut cake ***	Summer	382	47.0* (38.2-56.0)	303	1.9 (0.7-5.4)	N/A	
		Rainy season	382	23.1 (17.9-29.3)	303	1.7 (0.5-5.3)		
		Winter	382	27.2 (21.3-33.9)	303	1.7 (0.5-5.3)		
6.	Provision of sesame cake***	Summer	382	54.9* (46.2-63.2)	303	1.4 (0.4-4.7)	N/A	
		Rainy season	382	27.7 (22.2-34.1)	303	1.1 (0.3-4.8)		
		Winter	382	28.0 (22.2-34.7)	303	1.1 (0.3-4.8)		
7.	Provision of maize or sorghum straw	Summer	382	67.4* (63.4-71.1)	303	2.3 (0.8-6.1)	N/A	
		Rainy season	382	55.5 (50.9-60.0)	303	2.3 (0.8-6.1)		
		Winter	382	58.3 (53.1-63.3)	303	2.0 (0.7-5.5)		
8.	Free range scavenging	Summer	N/A		N/A		327	88.7 (80.8-93.6)
		Rainy season					327	90.6 (82.9-95.1)
		Winter					327	90.2 (83.1-94.5)
9.	Provision of rice	Summer	N/A		N/A		327	88.7 (83.2-92.6)
		Rainy season					327	90.8 (86.4-93.9)
		Winter					327	92.3 (88.0-95.2)
10.	Provision of broken rice	Summer	N/A		N/A		327	10.7 (6.4-17.3)
		Rainy season					327	10.0 (5.8-16.5)
		Winter					327	9.7 (5.6-16.2)
11.	Provision of peas	Summer	N/A		N/A		327	6.3 (3.0-12.6)
		Rainy season					327	6.1 (2.8-12.7)
		Winter					327	5.8 (2.6-12.4)
12.	Provision of household scrap	Summer	N/A		N/A		327	47.7 (38.8-56.8)
		Rainy season					327	45.7 (38.1-53.5)
		Winter					327	47.8 (39.5-56.2)
13.	Provision of maize	Summer	N/A		N/A		327	25.7 (19.1-33.7)
		Rainy season					327	22.9 (17.3-29.7)
		Winter					327	24.3 (17.9-32.0)
14.	Provision of water	Not provided	382	4.7 (2.7-8.0)	303	4.6 (2.3-8.9)	327	13.3 (8.2-20.8)
		River		0.9 (0.1-5.8)		2.8 (1.1-7.0)		1.0 (0.2-6.5)
		Well		78.6 (71.1-84.6)		68.1 (60.5-74.8)		69.7 (59.3-78.4)
		Lake		12.0 (7.4-18.9)		14.5 (9.7-21.1)		6.3 (3.5-11.0)
		Tap water		0.9 (0.3-2.9)		2.1 (0.7-6.6)		1.5 (0.4-5.9)
		Other		2.9 (1.7-5.2)		8.0 (4.9-12.8)		8.3 (4.7-14.1)

Summer = March-May; Rainy season = June-October; Winter = November-February

*Significant at p<0.05 for comparison of seasonal effects

**By-products of first-stage of processing harvested plants i.e., threshing and winnowing

***By-products of second-stage of processing harvested plants i.e. usually left over from oil extraction.

N/A Not available

Ruminants were generally provided with some form of shelter structure (cattle 82.2%; small ruminants 93.0%), while only 12.8% of farmers provided shelters to village chicken. A larger proportion of cattle (82.2%) and small ruminant farmers (93.0%) provided overnight shelters for animals. A large proportion of cattle and small ruminants provided shelter with natural material whereas the provision of shelter to village chicken was scarce (Supplementary Table 2). However, housing was more likely to be provided to cattle and small ruminants when they were kept alone, rather than in combination with other species (*p* = 0.058 for cattle; *p* = 0.0218 for small ruminants; Table 1).

Amongst ruminant-owning households, 56.8% (217 of 382) cattle households and 89.8% (272 of 303) small ruminant households used some form of breeding management. Cattle households commonly (86.7% or 188 of 217) used a bull from outside the household for mating, but was very common amongst small ruminant owners (87.1% or 237 of 272). Of 217 cattle owners, 56.7% used a bull from the same village for breeding, 27.7% used bulls from other villages and 1.8% used both their own bull and a bull from other villages while 13.3% had no active mating management. In contrast, of the 272 small ruminant farmers, 11.8% used a male from the same village, and 1.1% used a male from other villages whereas the rest of the farmers (87.1%) largely relied on males from within their own herd. Only 0.5% of cattle farmers used artificial insemination (AI), while no AI was conducted in small ruminants.

Castration was more common in cattle households (64.9%, 227 out of 342) compared to small ruminant households (5.0%, 18 out of 297). Usually, older cattle were castrated, with 97.4% older than 12 months at the time of castration and only 1.4% and 1.2% at 6–12 months and < 6 months, respectively. Out of the 18 small ruminant farmers practicing castration, 49.6% conducted castrations in animals older than 12 months, while 34.2% at 6–12 months and 16.2% at younger than 6 months.

### Husbandry characteristics associated with purpose of cattle rearing

Univariate analysis results for purpose of rearing are shown in Supplemanarty Table 4. However, in the final multinominal multivariable model, there was only an association between the purpose(s) of keeping cattle and cattle grazing. Grazing was more common for cattle kept for multiple purposes (OR 7.3, 95% CI 3.6–15.0) or exclusively for cash sales (OR 6.9, 95% CI 2.2–22.3) (*p* < 0.01) compared to cattle kept for agriculture focus (i.e. draught purposes and production of manure for fertiliser). Predicted probabilities for practising grazing across the three purposes of cattle rearing are shown in Supplementary Figure 1.

### Husbandry characteristics associated with herd or flock size

Larger cattle herds were more likely to practice grazing (*p* < 0.001) and to employ labour from outside the household to manage cattle than medium or small herds (*p* = 0.03; Table 3). In addition, larger cattle herds were more likely to be raised for multiple purposes (draught power production of fertiliser combined with sale of offsrig) compared to the sale of offspring alone (*p* < 0.05). Amongst small ruminant households, larger herds/flocks were kept by farmers with longer experience of small ruminant ownership (*p* = 0.003). Farmers keeping larger small ruminant flocks were more likely to use their own males for breeding, rather than males from other flocks (*p* < 0.001). For village chickens, only the provision of drinking water to birds was associated with larger flock sizes (*p* = 0.045).

**Table 3:** Final models of factors associated with the herd/flock size of cattle, small ruminants and village chickens in the CDZ of Myanmar

Variables	Categories	Number	Percentage (%)			OR	*p* value	Wald test
			Low	Medium	High			
Outcome variable: cattle herd size Low (1–3 heads)—156 (40.9%) Medium (4–6 heads)—130 (34.0%) High (> 6 heads)—96 (25.1%)								
Purpose of rearing	Cash commodity	382	56.9	57.7	22.1	1		0.0001
	Agriculture focus		21.1	32.8	37.0	1.2 (0.4–3.6)	0.685	
	Multipurpose		22.1	9.6	40.9	4.2 (1.8–9.9)	0.002	
Hire labour	No	382	91.0	83.7	76.0	1		–
	Yes		9.0	16.3	24.0	2.1 (1.1–4.0)	0.030	
Practice grazing	No	382	39.7	21.1	1.3	1		–
	Yes		60.3	78.9	98.7	4.3 (2.0–9.5)	0.000	
Outcome variable: small ruminant herd size Low (1–20 heads)—100 (33%) Medium (21–40 heads)—127 (41.9%) High (> 40 heads)—76 (25.1%)								
Duration of practising goat production	< 5 years	303	66.5	54.8	29.9	1		–
	> 5 years		33.5	45.2	70.1	3.0 (1.5–6.2)	0.003	
Provision of housing	No	303	19.5	2.8	1.1	1		–
	Yes		80.5	97.2	98.9	5.2 (1.1–24.4)	0.037	
Materials used for fencing	None	303	53.7	14.1	10.9	1		0.0008
	Bamboo		29.1	49.5	55.1	4.0 (1.4–11.7)	0.011	
	Wood		12.6	16.3	14.1	2.1 (0.7–6.1)	0.192	
	Plastic sheet		4.6	20.1	20.0	5.0 (1.7–14.5)	0.004	
Way of breeding	Own male	303	70.8	93.8	99.2	1		–
	Other male		29.2	6.2	0.8	0.1 (0.1–0.3)	0.000	
Outcome variable: village chicken flock size Low (1–7 heads)—115 (35.2%) Medium (8–14 heads)—98 (30%) High (> 14 heads)—114 (34.9%)								
Provision of water	Not provided	327	28.1	21.5	14.6	1		–
	Provided		71.9	78.5	85.4	1.8 (1.0–3.3)	0.045	

## Discussion

This study describes current livestock production systems in Myanmar and, importantly, identifies how different livestock enterprises interact with each other within a household. Existing studies frequently focus on a single livestock species and do not evaluate associations between livestock enterprises and thus may miss constraints or synergies faced by households owning multiple kinds of livestock (al-Naeem et al. 2000; Dreyer et al. 1999; Henning et al. 2007).

As in many farming systems worldwide and particularly in the developing world, our study highlights that most of the small-scale farmers in the CDZ of Myanmar keep more than one species of animal (Amenu et al. 2013; LIFT 2014; Maass et al. 2012). Our study also demonstrates that raising of village chickens in combination with cattle or small ruminants was more common than the combination of small and large ruminants, probably because chickens are managed easily, and do not compete for ruminant resources. Although we do not ask the reason of practicing, nonetheless multispecies rearing may also have a number of benefits such as reducing economic risk associated with keeping single livestock enterprise and supporting other agricultural enterprises such as draught power for cultivating and land preparation (Devendra and Thomas 2002b). In addition, optimising the use of husbandry resources such as sharing animal housing, raising multiple livestock livestock species such as raising village chicken with other livestock species is likely to spread of the usage of scarce resources. However, raising multispecies will be challenging because farmers might not have finances and time to raise multiple speices in their farm, in particular poorer or smaller households with limited resources.

One interesting finding from our study is that there was no significant changes in herd size in the cattle farmers and village chicken farmers with more experience of farmers on raising these animals, whereas the dramatic expanding small ruminant herd size was seen in farmers with more experience. One explanation might be the majority of the farmers raised cattle for supporting other income source (such as cropping) and chicken for source of protein source for household and the expanding of herd size for direct income such as sale might not be a major concern. On the other hand, small ruminant was mainly raised for direct income source and farmers might be aware of the benefit of raising small ruminant due to increasing market demand.

Overall, the animal management and husbandry factors identified in this study reflect the small-scale, dryland agriculture and livestock production that is practiced under the harsh environmental conditions of the CDZ. The region is characterised by a low annual average rainfall of around 600 mm, which restricts the growth of fodder plants and crops, and leads to seasonal periods of feed shortage for multiple species of animals (FAO 2011b). Thus, feed availability is a major constraint for livestock production in the CDZ and farmers have to address this through specific grazing practices and the provision of supplementary feed.

Larger cattle herds were more likely to practise grazing. A number of studies have shown that addtional time and labour is required to built larger livestock enterprises (Budisatria et al. 2007; Kristjanson et al. 2004b; Morand-Fehr and Boyazoglu 1999), and our findings are consistent with these studies. The provision of freshly cut grass and potentially also supplementary feed is expensive for farmers and therefore owners of larger cattle herds prefer the practice of grazing cattle. The use of additional labour might be a challenge for cattle farmers as labour migration and and therefore decreased labour availability has been highlighted as considerable constrain to livestock production in the CDZ (Kempel 2013; Phyo et al. 2016). Where cattle were used for draught power for crop production, farmers were more likely to actively manage animal nutrition, such as providing supplementary or full feeding to cattle at home.

However, our findings also indicate that shelters were more likely to be provided to larger sheep and goat herds compared to smaller herds. This could be due to the fact that sheep and goats of larger numbers need to be managed more efficiently and also represent a more substantial monetary value. Small ruminants were usually only grazed, despite their additional nutritional requirements which should have resulted in the provision of supplementary feed by farmers.

Although dry and hot weather conditions are common in the CDZ, drinking water was mainly provided to larger village chicken flocks. Even though the reason is not clear, one possible explanation might be that in households with small flock sizes, village chicken might be mainly kept for home consumption and ‘pocket money’ and therefore are not provided with the same level of adequate care as larger flocks. Provision of supplementary feed to village chickens is costly and is probably only justified when larger flocks are raised or village chickens are produced under semi-intensive farm conditions (Henning et al. 2007, 2008, 2013).

Our results showed that while in cattle farms outbreeding was common, inbreeding dominated small ruminant production. This presents a significant constraint to small ruminant production in light of a number of studies reporting poor performance being associated with the practice of inbreeding (Fahmy and Shrestha 2000; Hermas et al. 1987; Muasya et al. 2006). However, the direct effect of inbreeding, in particular impacts on body condition score, needs to be further investigated. Sheep and goat farmers seem to be unaware of benefits of outbreeding, or poor animal performance due to inbreeding might not be important for the sale of animals. Highlighting the benefits of outbreeding on cattle farms might be able to convince small ruminant farmers to change their breeding practices.

Although our study is the first to describe livestock husbandry practices in the CDZ of Myanmar, it also had a number of limitations. Firstly, data were collected on a memory recall by farmers which might affect the precision of the data collected. Secondly, herd and flock size information was collected for a single time point, which might not allow us to identify the seasonal variation of herd and flock sizes. And finally, our study mainly focused on the most common livestock species in CDZ, namely cattle, small ruminants and village chickens, but other livestock such as pigs and ducks are also raised in this areas (FAO 2011a).

## Conclusions

Our study has shown that multispecies rearing by households is common in Myanmar’s CDZ and species-specific husbandry practices are implemented by farmers to reduce nutritional and health stresses. Although, some practices that are beneficial for one livestock species (e.g. supply of supplementary feed, provision of shelters and outbredding) are seldom applied to other species within the same household, despite the benefits these would likely bring. This highlights the need to evaluate the household’s entire livestock production ‘system’ and that extension training should consider all livestock species raised in a household. Furthermore, under the harsh climatic conditions in the CDZ, the provision of grazing areas and water are the biggest challenges to livestock farmers. Policy makers have to consider these constraints identified in this study to develop guidelines for sustainable livestock production and livestock-based food security in the CDZ of Myanmar.

## Electronic supplementary material

ESM 1 (DOCX 30 kb)

## Abbreviations

*CDZ*: Central dry zone
*UQ*: The University of Queensland
*FMD*: Foot and mouth disease
*ND*: Newcastle disease
*ACIAR*: Australian centre for international agriculture research
*LBVD*: Livestock breeding and veterinary department
*PSUs*: Primary sampling units
*SSUs*: Secondary sampling units
*VCE*: Variance estimation
*FPC*: Finite population correction
*mm*: Millimetre
*IQR*: Interquartile rate
*μ*: Mean value
*CI*: Confident interval
*HH*: Household
*AI*: Artificial insemination
