# Supplementary material for: Characteristics of Livestock Husbandry and Management Practice in the Central Dry Zone of Myanmar
Source: Trop Anim Health Prod. 2018 Oct 30;51(3):643–54. doi: 10.1007/s11250-018-1738-9 (PMC6513792; doi:10.1007/s11250-018-1738-9)
Supplement: Supplementary file 1 — (DOCX 30 kb) [file 11250_2018_1738_MOESM1_ESM.docx]

# **SUPPLEMENTARY MATERIALS**

Supplementary figure 1 Predicted probabilities (95% confidence intervals) for purposes of rearing cattle and practising or not practising grazing in the CDZ of Myanmar

Supplementary Table 1. Experience of farmers raising livestock species in the CDZ of Myanmar

| **Species** | **Total number (N)** | **Proportion of farmers with 95% CI** | | |
| --- | --- | --- | --- | --- |
|  |  | **<5 years** | **5-10 years** | **>10 years** |
| Cattle | 382 | 9.2 (6.4-13.2) | 12.2 (8.1-18.0) | 78.6 (72.8-83.4) |
| Sheep | 303 | 87.2 (77.9-92.9) | 5.2 (2.4-10.9) | 7.7 (4.1-13.9) |
| Goats | 303 | 51.2 (43.1-59.2) | 19.6 (14.5-25.9) | 29.3 (22.0-37.7) |
| Village chickens | 327 | 23.9 (17.8-31.2) | 10.6 (6.8-16.3) | 65.5 (57.8-72.4) |

Supplementary table 2 Characteristics of shelters provided to livestock species in the CDZ of Myanmar

| **Name of variables** | **Categories** | **Cattle** | | **Small ruminants** | | **Village chickens** | |
| --- | --- | --- | --- | --- | --- | --- | --- |
|  |  | **N** | Proportion with 95% CI | **N** | Proportion with  95% CI | **N** | Proportion with 95% CI |
| Provision of shelters | Yes | 382 | 82.2 (77.5-86.1) | 303 | 93.0 (89.2-95.5) | 327 | 12.8 (9.4-17.2) |
|  | No |  | 17.8 (13.9-22.5) |  | 7.0 (4.5-10.8) |  | 87.2 (82.8-90.6) |
| Materials used for roof of shelters | Not provided | 382 | 16.4 (12.1-21.8) | 303 | 6.4 (3.9-10.2) | 327 | N/A |
|  | Corrugated metal |  | 31.0 (22.5-40.9) |  | 16.1 (10.3-24.1) |  |  |
|  | Thatch leaves |  | 45.8 (37.6-54.4) |  | 59.2 (49.9-68.0) |  |  |
|  | Plastic sheet |  | 6.8 (3.6-12.6) |  | 18.3 (10.7-29.6) |  |  |
| Materials used for construct of fencing | No | 382 | 88.2 (84.0-91.4) | 303 | 24.3 (18.4-31.5) | 327 | N/A |
|  | Bamboo |  | 4.8 (2.9-7.9) |  | 45.3 (35.5-55.6) |  |  |
|  | Wood |  | 1.1 (0.3-3.6) |  | 14.7 (8.1-24.9) |  |  |
|  | Plastic sheet |  | 5.9 (3.8-9.0) |  | 15.7 (11.0-22.0) |  |  |
| Location where livestock is kept overnight | Separate building | 382 | 77.5 (70.3-83.3) | 303 | 86.8 (81.3-90.9) | 327 | 1.7 (0.7-4.4) |
|  | Tied on the tree |  | 12.4 (8.8-17.3) |  | 2.0 (0.6-6.9) |  | N/A |
|  | Under the farm house |  | 4.9 (2.8-8.5) |  | 7.3 (4.4-11.9) |  | 2.5 (1.1-5.5) |
|  | Extension of the house |  | 4.6 (2.2-9.3) |  | 2.5 (1.0-6.2) |  | 2.4 (1.0-5.9) |
|  |  |  |  |  |  |  |  |
|  | Tethered in the grazing areas |  | 0.6 (0.1-2.8) |  | 1.1 (0.4-3.5) |  | N/A |
|  | Resting in trees |  | N/A |  | N/A |  | 68.2 (61.1-74.5) |
|  | Sitting on the ground |  | 0.0 |  | 0.3 (0.0-1.5) |  | 15.5 (11.4-20.7) |
|  | Sitting under a bamboo coop |  | N/A |  | N/A |  | 9.7 (7.1-13.0) |

Supplementary table 3 Univariate analysis of factors associated with the herd/flock size of cattle, small ruminants and village chickens in the CDZ of Myanmar

| **Variables** | **Categories** | **N** | **Herd/Flock size (%)** | | | **OR** | **p-value** | **Wald test** |
| --- | --- | --- | --- | --- | --- | --- | --- | --- |
|  |  |  | **Low** | **Medium** | **High** |  |  |  |
| **Outcome variable: Cattle herd size**  Low (1-3 heads) - 156 (40.9%)  Medium (4-6 heads) - 130 (34.0%)  High (>6 heads) - 96 (25.1%) | | | | | | | | |
| Hire labour | No | 382 | 91.0 | 83.7 | 76.0 | 1 |  | - |
|  | Yes |  | 9.0 | 16.3 | 24.0 | 2.4 (1.3-4.4) | 0.009 |  |
| Practice grazing | No | 382 | 39.7 | 21.1 | 1.3 | 1 |  | - |
|  | Yes |  | 60.3 | 78.9 | 98.7 | 5.5 (3.1-9.8) | <0.0001 |  |

| **Outcome variable: Small ruminant herd size**  Low (1-20 heads) - 100 (33%)  Medium (21-40 heads) - 127 (41.9%)  High (>40 heads) - 76 (25.1%) | | | | | | | | | | |
| --- | --- | --- | --- | --- | --- | --- | --- | --- | --- | --- |
| Duration of practicing goat production | <5 years | 303 | 66.5 | 54.8 | 29.9 | | 1 |  | | - |
|  | >5 years |  | 33.5 | 45.2 | 70.1 | | 2.9 (1.5-5.4) | 0.002 | |  |
| Provision of housing | No | 303 | 19.5 | 2.8 | 1.1 | | 1 |  | | - |
|  | Yes |  | 80.5 | 97.2 | 98.9 | | 11.2 (3.9-32.3) | <0.0001 | |  |
| Materials used for fencing | None | 303 | 53.7 | 14.1 | 10.9 | | 1 |  | | <0.0001 |
|  | Bamboo |  | 29.1 | 49.5 | 55.1 | | 6.6 (3.1-14.3) | <0.0001 | |  |
|  | Wood |  | 12.6 | 16.3 | 14.1 | | 4.7 (2.0-11.1) | 0.001 | |  |
|  | Plastic sheet |  | 4.6 | 20.1 | 20.0 | | 8.6 (3.8-19.4) | <0.0001 | |  |
| Place of housing | Share the same shelter with farmers | 303 | 17.6 | 8.7 | 3.6 | | 1 |  | | 0.0156 |
|  | Separate building |  | 78.1 | 89.4 | 96.4 | | 3.2 (1.5-7.1) | 0.004 | |  |
|  | Tethering |  | 4.3 | 1.9 | 0.0 | | 0.7 (0.2-1.8) | 0.403 | |  |
| Way of breeding | Own male | 272 | 70.8 | 93.8 | 99.2 | | 1 |  | | - |
|  | Other male |  | 29.2 | 6.2 | 0.8 | | 0.1 (0.0-0.3) | <0.0001 | |  |
| **Outcome variable: Village chicken flock size**  Low (1-7 heads) - 115 (35.2%)  Medium (8-14 heads) - 98 (30%)  High (>14 heads) - 114 (34.9%) | | | | | | | | | | |
| Provision of water | Not provided | 327 | 28.1 | 21.5 | | 14.6 | 1 |  | - | |
|  | Provided |  | 71.9 | 78.5 | | 85.4 | 1.8 (1.0-3.3) | 0.045 |  |  |

Supplementary table 4 Univariate analysis for factors associated with the purposes of raising cattle in the CDZ of Myanmar

| **Variables** | **Categories** | **N** | **Agriculture focus** | **Cash commodity** | | | **Multipurpose** | | | |
| --- | --- | --- | --- | --- | --- | --- | --- | --- | --- | --- |
|  |  |  | **%*** | **%*** | **RRR** | **p-value** | **%*** | **RRR** | **p-value** | **Wald test** |
| **Outcome variable: Purpose of rearing cattle**  Cash commodity - 52 (15.7%)  Agriculture focus - 111 (33.5%)  Multipurpose - 168 (50.8%**)** | | | | | | | | | | |
| Main income source | Cropping | 318 | 63.5 | 16.8 | 1 |  | 54.3 | 1 |  | 0.0037 |
|  | Livestock production |  | 14.7 | 38.7 | 10.0  (3.2-31.1) | <0.0001 | 18.1 | 1.5  (0.6-3.3) | 0.368 |  |
|  | Labour |  | 6.3 | 26.1 | 15.7  (3.7-66.8) | <0.0001 | 10.6 | 2.0  (0.8-4.7) | 0.122 |  |
|  | Shop owner |  | 1.0 | 6.7 | 24.8  (4.8-129.6) | <0.0001 | 5.1 | 5.9  (1.1-32.5) | 0.043 |  |
|  | Supported by relatives |  | 14.6 | 11.7 | 3.0  (1.0-9.7) | 0.062 | 11.93 | 1.0  (0.4-2.3) | 0.905 |  |
| Providing of housing | No | 331 | 9.3 | 38.4 | 1 |  | 12.8 | 1 |  | - |
|  | Yes |  | 90.7 | 61.6 | 0.2 (0.1-0.5) | 0.002 | 87.2 | 0.7 (0.2-2.2) | 0.530 |  |
| Materials used for roof of housing | Not provided | 331 | 6.3 | 38.4 | 1 |  | 11.8 | 1 |  | 0.0046 |
|  | Corrugated metal |  | 34.3 | 21.3 | 0.1 (0.0-0.3) | <0.0001 | 37.1 | 0.6 (0.2-1.9) | 0.352 |  |
|  | Thatch leaves |  | 49.9 | 32.5 | 0.1 (0.0-0.4) | 0.001 | 44.8 | 0.5 (0.2-1.5) | 0.197 |  |
|  | Plastic sheet |  | 9.4 | 7.8 | 0.1 (0.0-0.8) | 0.026 | 6.4 | 0.4 (0.1-1.7) | 0.195 |  |
| Practice grazing | No | 331 | 43.4 | 1.7 | 1 |  | 4.7 | 1 |  | - |
|  | Yes |  | 56.6 | 15.1 | 6.9 (2.2-22.3) | 0.002 | 44.9 | 7.3 (3.6-15.0) | <0.0001 |  |
| Provision of supplementary feed | No | 331 | 7.0 | 49.1 | 1 |  | 10.4 | 1 |  | - |
|  | Yes |  | 93.0 | 51.0 | 0.1 (0.02-0.3) | <0.0001 | 89.6 | 0.7 (0.3-1.5) | 0.301 |  |
| Practice castration | Not practice | 331 | 23.5 | 88.9 | 1 |  | 34.7 | 1 |  | - |
|  | Practice |  | 76.5 | 11.1 | 0.0 (0.0-0.1) | <0.0001 | 65.3 | 0.6 (0.3-1.4) | 0.206 |  |
| Cattle herd size | Low | 331 | 57.7 | 56.9 | 1 |  | 22.1 | 1 |  | <0.0001 |
|  | Medium |  | 32.8 | 21.1 | 0.7 (0.3-1.4) | 0.270 | 37.0 | 2.9 (1.7-5.1) | <0.0001 |  |
|  | High |  | 9.6 | 22.1 | 2.3 (0.6-9.4) | 0.223 | 40.9 | 11.1  (3.7-33.5) | <0.0001 |  |
